# Genome-Wide Identification and *in Silico* Analysis of Poplar Peptide Deformylases

**DOI:** 10.3390/ijms13045112

**Published:** 2012-04-23

**Authors:** Chang-Cai Liu, Bao-Guang Liu, Zhi-Wei Yang, Chun-Ming Li, Bai-Chen Wang, Chuan-Ping Yang

**Affiliations:** 1 State Key Laboratory of Forest Genetics and Tree Breeding, Northeast Forestry University, 26 Hexing Road, Harbin 150040, China; E-Mail: wbc007@163.com; 2 Laboratory for Chemical Defense and Microscale Analysis, P.O. Box 3, Zhijiang 443200, China; E-Mail: liuchangcai_0@126.com; 3 Forestry College, Beihua University, Jilin 132013, China; E-Mail: liubaoguang2005@163.com; 4 School of Basic Medical Sciences, Jiamusi University, Jiamusi 154000, China; E-Mail: yzws-123@163.com; 5 Forestry Research Institution of Heilongjiang Province, Harbin 150081, China; E-Mail: lichunming_lcm@163.com

**Keywords:** peptide deformylase, *N*-terminal Met excision, *in silico* simulation, genome-wide investigation, phylogenetic analysis, gene duplication, ghromosome location, gene structure display

## Abstract

Peptide deformylases (PDF) behave as monomeric metal cation hydrolases for the removal of the *N*-formyl group (Fo). This is an essential step in the *N*-terminal Met excision (NME) that occurs in these proteins from eukaryotic mitochondria or chloroplasts. Although PDFs have been identified and their structure and function have been characterized in several herbaceous species, it remains as yet unexplored in poplar. Here, we report on the first identification of two genes (*PtrPDF1A* and *PtrPDF1B*) respectively encoding two putative PDF polypeptides in *Populus trichocarpa* by genome-wide investigation. One of them (XP_002300047.1) encoded by *PtrPDF1B* (XM_002300011.1) was truncated, and then revised into a complete sequence based on its ESTs support with high confidence. We document that the two *PDF1s* of *Populus* are evolutionarily divergent, likely as a result of independent duplicated events. Furthermore, *in silico* simulations demonstrated that PtrPDF1A and PtrPDF1B should act as similar PDF catalytic activities to their corresponding PDF orthologs in Arabidopsis. This result would be value of for further assessment of their biological activities in poplar, and further experiments are now required to confirm them.

## 1. Introduction

In all organisms, the protein synthesis machinery requires newly synthesized peptides to start systematically with methionine (Met) [1]. Approximately two-thirds of mature proteins undergo *N*-terminal Met excision (NME) by Met aminopeptidase (MAP; EC 3.4.11.18), which proteolytically removes the *N*-terminal Met if the residue at position two has a side-chain with a radius of gyration of 1.29 Å or less [2–5]. However, MAP cannot cleave Met with an *N*-formyl group (Fo) from eubacteria, mitochondria and chloroplasts, where the *N*-terminal Met moiety must be *N*-formylated by a formyltransferase [5,6].

Removal of the Fo is undertaken by peptide deformylase (PDF), and is therefore an essential first step in allowing the subsequent NME occurrence in the eukaryotic mitochondria or chloroplasts [1,2,7]. Most PDFs are monomeric hydrolases and all contain three signature sequence motifs, comprising the active pocket of the enzyme and a metal cation: (i) G*ψ*G*ψ*AAXQ (motif 1); (ii) EGCLS (motif 2) and (iii) HE*ψ*DH (motif 3), where *ψ* is a hydrophobic amino acid [8,9]. The Cys of motif 2 and the two His residues of motif 3 stabilize metal ion coordination at the active site of PDF [8,9]. PDFs are important for some biological processes such as development of chloroplast in rice [10], and cell proliferation in humans [11].

Recent studies, together with the release of complete genome sequences for different organisms, have led to the identification of PDFs in eukaryotes; two PDFs have been identified in Arabidopsis [8,9], three in rice [10] and one in humans [11]. Since these PDFs do not contain the two insertions typical of PDF2 molecules, all eukaryotic PDFs are grouped as type 1 (PDF1). In Arabidopsis, the two PDF1s form two subclasses: PDF1A that localizes to the mitochondria, and PDF1B that localizes to plastids [1,12]. These crystal structures of Arabidopsis PDF1A and PDF1B have been determined, not only indicating several similarities to bacterial PDFs and their function activity for the removal of the *N*-formyl group, but also showing several clear differences between AtPDF1A (At1g15390.1) and AtPDF1B (At5g14660.1) [8,9].

Although amount of research efforts have been employed in exploring PDFs structure and function for several plant species, such as Arabidopsis [1,8,9,12] and rice [10], such research has not yet been directed towards woody trees. In order to identify all genes encoding PDFs and explore their function in poplar, we initiate one genome-wide investigation combined with *in silico* simulations. In this work, we identified two genes encoding PDFs across the complete *P. trichocarpa* genome, and proposed that poplar PDFs should possess similar biological activities to their corresponding PDF orthologs in Arabidopsis. This result would be valuable towards further assessment of their functional roles in poplar.

## 2. Results and Discussion

### 2.1. Identification and Characterization of *PDF* Genes in Populus

To identify poplar *PDF* genes and their putative encoded polypeptides occurred in the complete *P. trichocarpa* genome, Hidden Markov Model (HMM) profile file of the PDF domain (PF01327) [13,14] was exploited as a query file for a search across the *P. trichocarpa* protein sequence data [15]. A total of two non-redundant putative genes were identified as *PDF* genes because of their encoding polypeptides significantly matched the known PDF domain (Table 1). Furthermore, to calibrate our identification of the two *PDF* genes from JGI poplar database, their encoding proteins were further compared by a BLASTP search against NCBI Reference sequence (RefSeq) database, which provides a non-redundant and validated collection of sequences representing genomic data, transcripts and proteins [16,17]. As a result, the two poplar *PDF* genes (640630 and 173925) respectively possess their individual counterparts of protein and mRNA in NCBI RefSeq database (Table 1), suggesting that they should represent correct proteins or genes. Thus, in this endeavor, two *PDF1* genes (and their corresponding encoding PDF proteins) were identified in total across the *P. trichocarpa* genome by the genome-wide investigation. The *P. trichocarpa* genome encodes the similar numbers of *PDF1* gene members as several herbaceous plants, such as Arabidopsis [12] and rice [1], indicating no expansion present in poplar *PDF* gene members. In contrast, the expansion was often present in large number of *Populus* multigene families [15]. The result might reflect the analogous need for PDF activities involved in Fo Removal between woody and herbaceous plants.

### 2.2. Revision of Poplar *PDF* Gene-encoding Proteins

**Figure 1:**
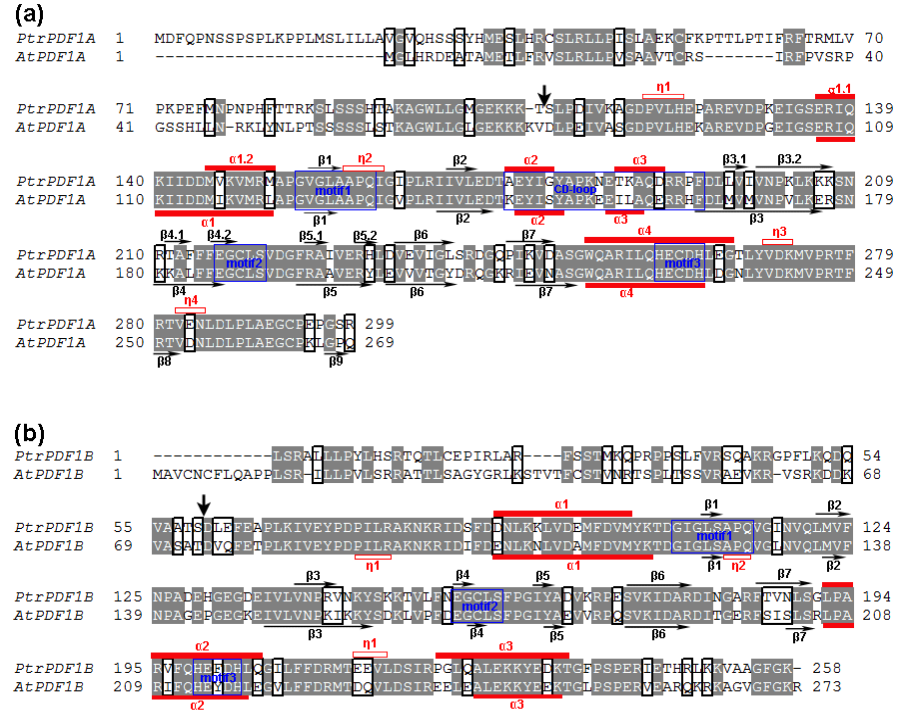
Alignment of the PDF sequences between poplar and Arabidopsis. One complete amino acid sequence alignment of the two poplar PDFs with their orthologs in Arabidopsis was performed. It was found that they respectively shared the best amino acid sequence identities with AtPDF1A (AT1G15390) and AtPDF1B (AtPDF1B). Motifs 1, 2 and 3 are indicated as blue frames. White characters in grey boxes indicate strict identity, and black characters in white boxes indicate similarity. α, η and β represent α-helix, short 3_10_ helix and β-sheets, respectively. (**a**) Sequence alignment of PtrPDF1A (XP_002298107.1) with AtPDF1A of Arabidopsis; (**b**) Sequence alignment of PtrPDF1B (XP_002300047.1) with AtPDF1B of Arabidopsis. Gaps were introduced to insure maximum identity.

**Figure 2:**
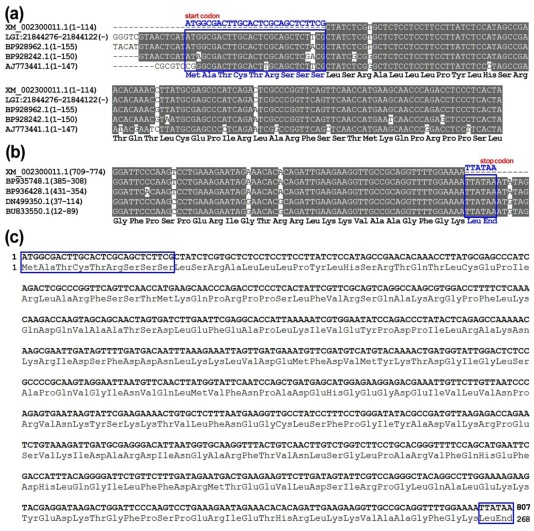
Revision of *PtrPDF1B* transcript and its encoding protein from NCBI RefSeq database by multiple sequence alignment. (**a**) Multiple sequence alignment of 5′ terminus between the original *PtrPDF1B* transcript, as well as its corresponding genome DNA and ESTs; (**b**) Multiple sequence alignment of 3′ terminus between the original *PtrPDF1B* transcript and its corresponding ESTs; (**c**) Schematic diagram of the revised complete CDS of *PtrPDF1B* and its encoding full-length protein sequence. The amino acid encoded by each codon is displayed in the bottom of sequence alignment. Nucleotide acid sequences marked with open blue box represents the extended 5′ or 3′ terminus of *PtrPDF1B* transcript, while amino acid sequences marked with open blue box represents the extended *N*- or *C*- terminus of PtrPDF1B protein.

To provide a simplified nomenclature for each identified protein, the two identified PDFs were respectively denominated as *PtrPDF1B* (XP_002300047.1) and *PtrPDF1A* (XP_002298107.1) according to their individual best hits with their orthologs in Arabidopsis (Figure 1 and Table 1). It is noteworthy that the coding sequence (CDS, XM_002300011.1) encoding PtrPDF1B might be uncompleted because of its absence of start codon “ATG” and stop codon, which leads to the truncated *N*-terminus and *C*-terminus of PtrPDF1B proteins. In order to amend it (XM_002300011.1) into complete CDS sequence, its corresponding Expressed Sequence Tags (ESTs) were retrieved by a BLASTN online search [18]. These 5′ and 3′ perfectly matched ESTs from NCBI were respectively applied for the alignment with 5′ and 3′ terminus of the CDS sequence (Figure 2a,b). The sequence alignment and further comparative analyses clearly demonstrated that upstream of the first three nucleotides “CTA” from the transcript (XM_002300011.1) should be extended by the “ATG” encoding Met as initiation codon as well as the followed 24 nucleotide acid sequences encoding one polypeptide with 8 consecutive amino acids (Figure 2a). Furthermore, downstream of the last three nucleotides “AAA” from the transcript (XM_002300011.1) should be extended by the “TTA” encoding Leu as well as the following “TAA” encoding stop codon (Figure 2b). Although the CDS (XM_002300011.1) and protein sequence (XP_002300047.1) of *PtrPDF1B* were obtained from the NCBI Reference sequence (RefSeq) database, which provides a non-redundant and validated collection of sequences representing genomic data, transcripts and proteins [16,17], they will need to be refined since they could represent one truncated transcript or protein. In this endeavor, the truncated CDS/transcript of PtrPDF1B were confirmed by ESTs support with high confidence and revised into complete CDS sequence, whereas the corresponding full-length protein sequence of *PtrPDF1B* was also obtained, as shown in Figure 2a–c.

### 2.3. Divergence in Poplar *PDF1s*

**Figure 3:**
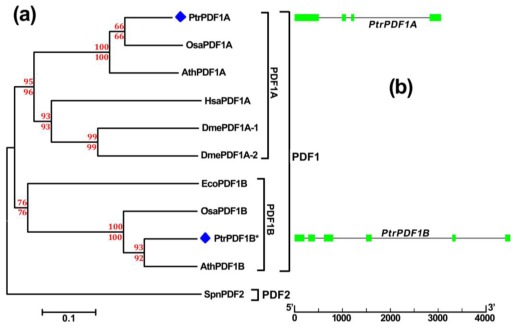
Phylogenetic analysis and gene structure display of the *Populus PDF1* genes (**a**) Phylogenetic analysis of *Populus PDF1* genes. Neighbor-joining bootstrap and Minimum Evolution values for clans supported above the 60% level were respectively indicated above and below the branches in red font. All PDF protein names and their individual corresponding ID number for phylogenetic analysis are listed as follows: SpnPDF2 (Q9F2F0); EcoPDF1B (P0A6K3); DmePDF1A-1 (Q8INL3); DmePDF1A-2 (Q9VGY2); HsaPDF1A (Q9HBH1); OsaPDF1B (Q5VNN5); OsaPDF1A (B6RGY0); AthPDF1B (Q9FUZ2); AthPDF1A (Q9FV53); PtrPDF1A (XP_002298107.1); PtrPDF1B (XP_002300047.1). The blue diamonds are highlighted in the front of all PtrPDF1A and PtrPDF1B from *Populus*. PtrPDF1B* represents the revised PtrPDF1B protein sequence in our study; (**b**) Schematic representation of the intron/exon structure for the Populus PDF1 genes. Exons and introns of Populus PDF1 genes are represented by green boxes and black lines, respectively, and their sizes could be estimated by the scale at the bottom.

Divergence in *PDF1s* that might give rise to be functionally distinct has found in herbaceous plants, such as Arabidopsis and rice. To examine whether similar PDF1s divergence occurs in *Populus*, an unrooted tree was constructed by both Neighbor-Joining [19] and Minimum-Evolution methods using MEGA 5.0 [20] based on alignments of these full-length PDF proteins sequences (Figure 3a). The tree topologies generated by the two methods were comparable without modifications at branches, and supported by their high bootstrap values of >60, suggesting that we constructed a reliable unrooted tree topology, in which two distinct clans occur, including *PDF1* and *PDF2* clans (Figure 3a). Phylogenetic analysis demonstrates that PDF1 of *Populus* is encoded by evolutionarily divergent genes, which is consistent with previous reports in *Arabidopsis* and rice (PDF1A and PDF1B; Figure 3a) [2]. In addition, divergence occurred between *PtrPDF1A* and *PtrPDF1B*. This is supported by an apparent difference in their amino acid sequences, especially with one relatively shorter *C*-terminal sequence in *PtrPDF1B*. Our results indicated that divergence of *PDF1* should be extended to *Populus* as a model woody plant, and the divergence might be caused by independent duplicated events. It is worth noting that another obvious divergence also exists in *PDF1A* (plant type *PDF1A* and animal type *PDF1A*) that the result supports previous phylogenetic analyses (Figure 3a) [2].

The gene structural display could provide us additional information for the evolutionary relationship of multi-gene families [21]. To further gain novel insight into the phylogenetic relationship of poplar *PDF1* genes, the exon/intron organization was illustrated for individual *PDF1* genes by comparison of the cDNA sequences and their corresponding genomic sequences (Figure 3b). As a result, the two evolutionarily divergent *PDF1* genes members in poplar exhibited a different distribution of exon/intron structure such that *PtrPDF1A* and *PrtPDF1B* respectively possessed four and six exons in their individual coding regions (Figure 3b). The difference in exon/intron architecture of *PtrPDF1A* and *PrtPDF1B* might support the divergence in *PDF1* genes of poplar from the phylogenetic analysis (Figure 3a).

### 2.4. Chromosome Location and Duplication of *PDF1* Genes in *Populus*

**Figure 4:**
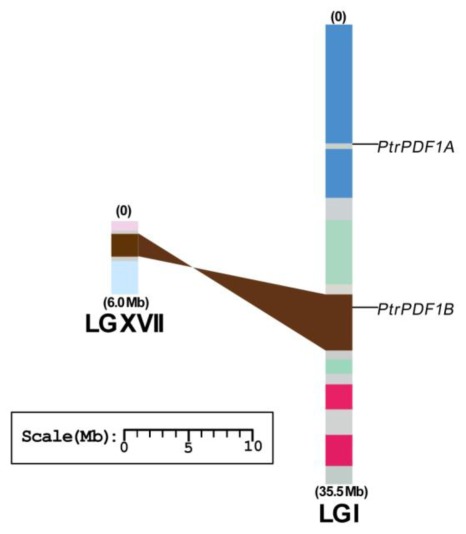
Chromosomal location of the *Populus PDF1* genes. Both two genes are mapped to the Linkage Groups I (LG I), one of nineteen LGs. Segmental duplicated homologous regions in the LG I and LG XVII of *Populus* obtained from the research of Tuskan and its co-workers [15], are shown with the common colors. The duplication blocks containing *PDF1* genes are connected with lines in shaded colors. Chromosome numbers (LG I and XVII) and sizes (Mb) are indicated at the bottom and end of each chromosome. Scale at the bottom represents a 10 Mb chromosomal distance.

*In silico* mapping of the gene loci showed that both the two *PDF* genes of *PtrPDF1A* and *PtrPDF1B* were found on Linkage Group I (LG I), one of the 19 LGs (Table 1 and Figure 4). Previous analysis of *Populus* genome has identified the presence of paralogous segments caused by the whole-genome duplication event in the Salicaceae (salicoid duplication), which occurred 65 million years ago and significantly contributed to the amplification of many multi-gene families [15]. To determine the possible relationship between the *PDF1* genes and paralogous segments, the *Populus PDF1* genes were mapped to the duplicated blocks of *P. trichocarpa* established in the studies of Tuskan and its coworkers [15]. The distribution of *PDF1* genes relative to the duplicated blocks is illustrated in Figure 4. It was found that *PtrPDF1B* gene (50%), are represented within duplicated blocks, whereas *PtrPDF1A* are outside these duplicated blocks, suggesting that their occurrence should be caused by independent duplication events. The result is surprisingly consistent with the deduction from our phylogenetic analysis above. Furthermore, one duplicated pair (*PtrPDF1B*) harbored *PDF1* genes on only one of the blocks and lack corresponding duplicates, suggesting that dynamic changes on the loss event of its corresponding paralogous genes might have occurred following segmental duplication (Figure 4). The findings support the result that the most abundant genes losses in eukaryotes occur following the whole genome duplication [22].

### 2.5. *In Silico* Simulation on the Poplar *PDFs* Reveal Analogous Activities with Their Individual Counterparts in Arabidopsis

**Figure 5:**
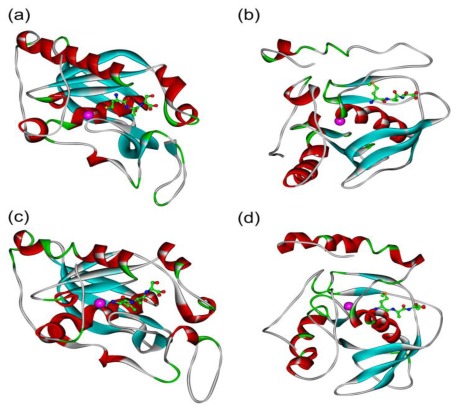
Ribbons stereo views. (**a**) Ribbons stereo views of AtPDF1A; (**b**) Ribbons stereo views of AtPDF1A; (**c**) Ribbons stereo views of PtrPDF1A; (**d**) Ribbons stereo views of PtrPDF1B. The substrate Met-Ala-Ser is represented by a ball and stick model. Zn^2+^ is in the purple CPK model. Ribbon colors: helices, β-sheets, turns and random coils are in red, cyan, green and white, respectively.

The sequence alignment of *PtrPDF1A* and *PtrPDF1B* with known PDF sequences from Arabidopsis separately revealed high sequence similarity, especially the three conserved function-related regions, motif 1, motif 2 and motif 3 (Figure 1a,b). Consequently, PDF activity should be present in the two identified *PtrPDFs* in poplar. However, high sequence homology of the primary structure only partly provides evidence for their analogous catalytic activity. The *in silico* modeling of *PtrPDF1A* and *PtrPDF1B* were performed to explore the functions of these two proteins. As Figure 5 shows, *PtrPDF1A* consists mainly of helices, β-sheets, turns and random coils (Figure 5c). It is identical to the structure of the known AtPDF1A (PDB code 1ZY1) protein [9], especially for the three conserved motifs (Figure 5a). However, there are differences in regions not directly related to the function. For example, the *N*-terminal α1-helix region of PtrPDF1A is split into two α-helices by a single turn whereas in AtPDF1A this is one continuous α1-helix. A similar situation is also observed between PtrPDF1B and AtPDF1B (PDB code 3CPM) [8] (Figure 5b,d).

**Figure 6:**
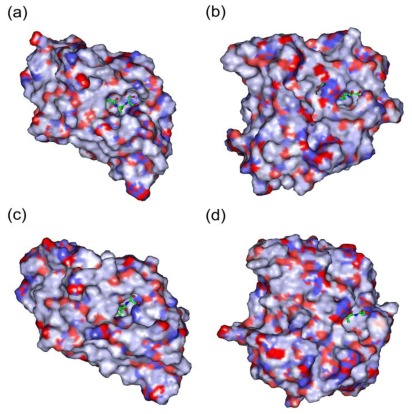
Surface electrostatic potential. (**a**) Surface electrostatic potential of AtPDF1A; (**b**) Surface electrostatic potential of AtPDF1B; (**c**) Surface electrostatic potential of PtrPDF1A; (**d**) Surface electrostatic potential of PtrPDF1B. The Connolly surfaces of the proteins were created using the InsightII 2005 scripts. The electrostatic potential is indicated by the color saturation (red for negative and blue for positive).

As discussed above, the structures of PtrPDF1A and PtrPDF1B are similar to AtPDF1A and AtPDF1B, respectively. This conclusion is further supported by the analysis of the electrostatic potential surfaces (EPS). It is clear that the active sites of PtrPDF1A and PtrPDF1B are nearly the same as those of AtPDF1A and AtPDF1B, respectively (Figure 6). In addition, the binding sites of the substrate Met-Ala-Ser within AtPDF1A and PtrPDF1A are close in the structure (Figure 6a,c). The interaction energies (*E*_inter_) were calculated to be −208.75 and −122.21 kcal mol^−1^, respectively. During the ligand binding processes electrostatic effects play a large role, which amounts to 79% and 60% of the binding energies, respectively. For AtPDF1B and PtrPDF1B (Figure 6b,d), the energy values were −199.31 and −222.30 kcal mol^−1^, respectively. Electrostatic interactions (*E*_ele_) rather than van der Waals interactions (*E*_vdW_) play a dominant role in the ligand binding processes, contributing to almost 79% and 85% of the binding energies, respectively. In particular, PtrPDF1A and PtrPDF1B recognize the tripeptide Met-Ala-Ser, which is consist with experiments and previous reports [8,9]. The results provide a hypothesis that the putative PDFs of poplar should act with PDF catalytic activity and in a similar mechanism to their corresponding PDF orthologs in Arabidopsis. This result is important for further studying and examining their biological activities.

## 3. Experimental Section

### 3.1. Identification of PDF Genes across Poplar Genome

The complete protein sequence database was downloaded from *Populus trichocarpa* v1.1 [23]. Hidden Markov Model (HMM) profile file (Pep_deformylase.hmm) of the Pfam PDF domain (PF01327) from the Pfam database [24], was exploited as a query file to identify *PDF* genes in the *Populus* protein database using the hmmer search command of the HMMER (v 3.0) program, which was widely applied for identification of homologues of an interested protein family [14,25].

### 3.2. Revision of Poplar PDF Proteins

The expressed sequence tags (EST) were retrieved by BLASTN the corresponding transcript/CDS from *P. trichocarpa* v1.1 [23] as query sequence online search against all of the *Populus* EST sequences in NCBI. Matches above 95% identity and over an alignment of at least 100 bp were considered as corresponding sequences of the *PDF* genes. Multiple sequences alignments of these sequences with their individual transcript/CDS sequence were performed using ClustalW program in BioEdit software under the default parameters settings [26]. Sequence alignments were manually adjusted to get maximum matching.

### 3.3. Phylogenetic Analysis and Gene Structural Display

The unrooted phylogenetic trees were constructed using MEGA 5.0 software [20], by both the Neighbor-joining method [19] and Maximum Likelihood method with parameters (*p*-distance and completed deletion) based on 11 aligned *PDF* sequences. The reliability of the phylogenetic tree was estimated using bootstrap value with 1000 replicates. Gene structure display server (GSDS) program [21] was applied to the illustrate exon/intron organization for individual *PDF* genes by comparison of the cDNA sequences and their corresponding genomic sequences.

### 3.4. Chromosomal Location and *in Silico* Simulation

The two identified *PDF* genes were located in the genome of *P. trichocarpa* using NCBI map viewer [27]. Identification of duplicated regions between chromosomes was completed as described in Tuskan *et al*. [15].

All the flexible docking simulations were performed with the different modules implemented under the InsightII 2005 software package [28] on Linux workstations, using the consistent-valence force-field (CVFF). The X-ray crystal structures AtPDF1A (PDB code 1ZY1) [9] and AtPDF1B (PDB code 3CPM) [8] were recovered from the RCSB Protein Data Bank and employed to construct the structures of PtrPDF1A and PtrPDF1B, applying the workspace in the Swiss Model [29,30]. The two protein models were optimized with the conjugated gradient algorithm (Discover 3.0 module). Geometry and partial atomic charges of the tripeptide Met-Ala-Ser were conducted throughout the Discover 3.0 module by applying the BFGS algorithm [31] with a convergence criterion of 0.01 kcal·mol^−1^·Å^−1^. As demonstrated by previous results [32,33], the docking simulations were performed to explore and understand the interactions of PtrPDF1A and PtrPDF1B with the tripeptide Met-Ala-Ser using the general protocols in the InsightII 2005 software packages [32,34]. The interaction energies of the substrate with proteins were calculated by the Docking module [34]. More details describing the calculation processes can be found elsewhere [32,33].

## 4. Conclusions

Removal of the Fo undertaken by *PDF* is an essential first step of the NME occuring in the eukaryotic mitochondria or chloroplasts. Some advances have been made in exploring structure and function of PDFs for several plant species, such as Arabidopsis, maize and rice. However, such effort has not yet been directed towards poplars as model woody trees. In this work, the above issues are addressed using the method of one genome-wide investigation combined with *in silico* simulations. *P. trichocarpa* genome contains two evolutionarily divergent genes of *PtrPDF1A* and *PtrPDF1B*, which might be caused by independent duplicated events. Furthermore, PtrPDF1A and PtrPDF1B should act with similar PDF catalytic activity to their corresponding PDF orthologs in Arabidopsis. These results would be valuable resources for understanding the function of PDFs in poplar, and further experiments, based on our results, should be performed in the future.

## Figures and Tables

**Table 1:** Characterization and identification of Peptide deformylases (PDF) genes of poplar.

JGI NO.	Novel simplified nomenclature	Refseq protein ID	Refseq RNA ID (CDS)	Chromosome Location
640630	PtrPDF1A	XP_002298107.1	XM_002298071.1	LG_I: 9208431–9211542 (+)
173925	PtrPDF1B	XP_002300047.1	XM_002300011.1	LG_I: 21839768–21844235 (−)
